# Association Between Social Vulnerability and a County’s Risk for Becoming a COVID-19 Hotspot — United States, June 1–July 25, 2020

**DOI:** 10.15585/mmwr.mm6942a3

**Published:** 2020-10-23

**Authors:** Sharoda Dasgupta, Virginia B. Bowen, Andrew Leidner, Kelly Fletcher, Trieste Musial, Charles Rose, Amy Cha, Gloria Kang, Emilio Dirlikov, Eric Pevzner, Dale Rose, Matthew D. Ritchey, Julie Villanueva, Celeste Philip, Leandris Liburd, Alexandra M. Oster

**Affiliations:** 1 CDC COVID-19 Response Team.

Poverty, crowded housing, and other community attributes associated with social vulnerability increase a community’s risk for adverse health outcomes during and following a public health event (*1*). CDC uses standard criteria to identify U.S. counties with rapidly increasing coronavirus disease 2019 (COVID-19) incidence (hotspot counties) to support health departments in coordinating public health responses (*2*). County-level data on COVID-19 cases during June 1–July 25, 2020 and from the 2018 CDC social vulnerability index (SVI) were analyzed to examine associations between social vulnerability and hotspot detection and to describe incidence after hotspot detection. Areas with greater social vulnerabilities, particularly those related to higher representation of racial and ethnic minority residents (risk ratio [RR] = 5.3; 95% confidence interval [CI] = 4.4–6.4), density of housing units per structure (RR = 3.1; 95% CI = 2.7–3.6), and crowded housing units (i.e., more persons than rooms) (RR = 2.0; 95% CI = 1.8–2.3), were more likely to become hotspots, especially in less urban areas. Among hotspot counties, those with greater social vulnerability had higher COVID-19 incidence during the 14 days after detection (212–234 cases per 100,000 persons for highest SVI quartile versus 35–131 cases per 100,000 persons for other quartiles). Focused public health action at the federal, state, and local levels is needed not only to prevent communities with greater social vulnerability from becoming hotspots but also to decrease persistently high incidence among hotspot counties that are socially vulnerable.

Daily county-level COVID-19 case counts were obtained through USAFacts (https://usafacts.org/), which compiles data reported by state and local health departments.* Beginning on March 8, 2020, hotspot counties were identified daily using standard criteria^†^ (*2*). County-level social vulnerability data were obtained from the 2018 CDC SVI, which was developed to identify communities with the most needs during and following public health events. Scores for overall SVI, along with four vulnerability subcomponents pertaining to 1) socioeconomic status, 2) household composition and disability, 3) representation of racial and ethnic minority groups and English proficiency, and 4) housing type and transportation, were generated using 15 population-based measures.^§^ Scores for the overall and subcomponent measures were presented as percentile rankings by county, with higher scores indicating greater vulnerability. SVI scores were categorized as quartiles based on their distribution among all U.S. counties. Urbanicity of counties was based on the National Center for Health Statistics 2013 urban-rural classification scheme^¶^ (*3*).

Counties meeting hotspot criteria at least once during March 8–July 25 were described by urbanicity and social vulnerability based on the first date of hotspot detection. All other analyses were limited to hotspots identified during June 1–July 25. Among all 3,142 U.S. counties, RRs with 95% CIs were calculated using bivariate log-binomial models to assess differences in the probability of being identified as a hotspot during June 1–July 25 by SVI quartile, overall and for the four SVI subcomponents; analyses were also stratified by urbanicity.** Based on these results, the probability of hotspot identification was further examined by specific measures of social vulnerability related to the representation of the following groups in each county: racial and ethnic minority residents, English proficiency, housing type, and transportation; counties were categorized as at or above or below the national median values.

Among the 747 counties meeting hotspot criteria during June 1–July 25, 689 (92%) were classified as new hotspots.^††^ Among these 689 counties, the median COVID-19 incidence^§§^ was calculated over the 14 days after hotspot identification and compared with incidence during the same period among 689 randomly selected non-hotspot counties matched by three urbanicity categories. Among new hotspot counties, incidence was also compared by SVI quartile.^¶¶^ All analyses were conducted using SAS (version 9.4; SAS Institute) and R (version 4.0.2; The R Foundation). P-values <0.05 were considered statistically significant.

The percentage of hotspots in nonmetropolitan areas increased from 11% during March–April to 40% during June–July (Figure 1). The percentage of hotspots in the highest SVI quartile increased from 22% during March–April to 42% during June–July (Figure 1).

**FIGURE 1:**
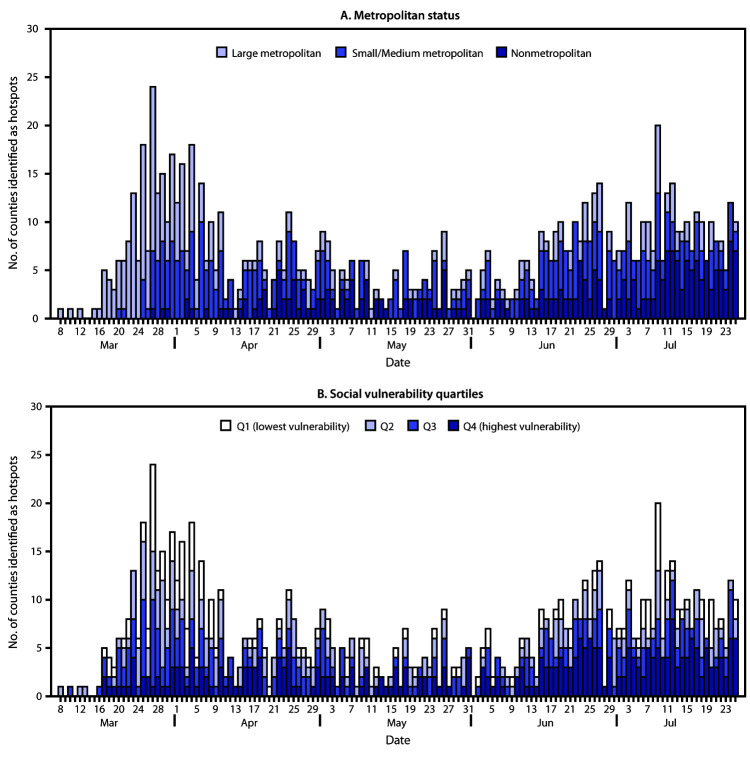
Daily number of counties identified as hotspots, by urbanicity (A)* and by quartiles of overall social vulnerability index score (B), based on first date of hotspot identification (N = 905 counties)^†^^,^^§^— United States, March 8–July 25, 2020 * According to the 2013 National Center for Health Statistics Urban-Rural Classification Scheme for counties, counties can be grouped into one of six categories based on population size, including large central metropolitan, large fringe metropolitan, medium metropolitan, small metropolitan, micropolitan, and noncore areas. For this analysis, results were presented in three categories: large central metropolitan and large fringe metropolitan (large metropolitan), medium and small metropolitan, and micropolitan and noncore areas (nonmetropolitan). ^†^ Overall social vulnerability scores were percentile rankings ranging from 0–1, with higher values indicating greater social vulnerability. Scores were categorized into quartiles based on distribution among all U.S. counties. ^§^ Each county only appears once and is represented based on the first date of hotspot identification during March 8–July 25, 2020.

During June 1–July 25, 747 (24%) U.S. counties (representing 60% of the U.S. population) were identified as hotspots (Table). Counties with higher social vulnerability, particularly vulnerabilities related to the representation of racial and ethnic minority residents, English proficiency, housing type, and transportation, had a higher probability of being identified as a hotspot. For example, the risk for becoming a hotspot was 37.3 (95% CI = 20.1–69.3) times as high among areas in the highest quartile of vulnerability related to representation of racial and ethnic minority residents and English proficiency and 3.4 (95% CI = 2.7–4.2) times as high among areas in the highest quartile of vulnerability related to housing type and transportation, compared with areas in the lowest quartile for these vulnerabilities. These vulnerability subcomponents were more strongly associated with hotspot identification in less urban areas. Counties with median percentage or higher of racial and ethnic minority residents (RR = 5.3; 95% CI = 4.4–6.4), housing structures with ≥10 units (RR = 3.1 [2.7–3.6]), and crowded housing units (i.e., more persons than rooms) (RR = 2.0; 95% CI = 1.8–2.3) were more likely to become hotspots.

**TABLE:** Associations between social vulnerability measures* and hotspot identification, overall and by urbanicity^†^ (N = 3,142 total counties) —United States, June 1–July 25, 2020

Social vulnerability	All counties			Large metropolitan counties			Medium and small metropolitan counties			Nonmetropolitan counties		
	Overall	Hotspots		Overall	Hotspots		Overall	Hotspots		Overall	Hotspots	
	No.	No. (row %)	RR (95% CI)^¶^	No.	No. (row %)	RR (95% CI)^¶^	No.	No. (row %)	RR (95% CI)^¶^	No.	No. (row %)	RR (95% CI)^¶^
**Overall (row %)**	**3,142**	**747 (24)**	**—**	**436**	**227 (52)**	**—**	**372**	**190 (51)**	**—**	**1,976**	**195 (10)**	**—**
**Overall social vulnerability**												
Q1 (lowest vulnerability)	786	109 (14)	Reference	171	68 (40)	Reference	152	34 (22)	Reference	463	7 (2)	Reference
Q2	784	176 (22)	1.6 (1.3–2.0)	122	68 (56)	1.4 (1.1–1.8)	205	96 (47)	2.1 (1.5–2.9)	457	12 (3)	1.7 (0.7–4.4)
Q3	785	198 (25)	1.8 (1.5–2.2)	99	59 (60)	1.5 (1.2–1.9)	212	98 (46)	2.1 (1.5–2.9)	474	41 (9)	5.7 (2.6–12.6)
Q4 (highest vulnerability)	786	263 (33)	2.4 (2.0–2.9)	44	32 (73)	1.8 (1.4–2.4)	161	97 (60)	2.7 (2.0–3.7)	581	134 (23)	15.3 (7.2–32.3)
**Social vulnerability related to socioeconomic status**												
Q1 (lowest vulnerability)	785	167 (21)	Reference	180	95 (53)	Reference	176	62 (35)	Reference	429	10 (2)	Reference
Q2	786	197 (25)	1.2 (1.0–1.4)	144	72 (50)	0.9 (0.8–1.2)	218	107 (49)	1.4 (1.1–1.8)	424	18 (4)	1.8 (0.9–3.9)
Q3	784	188 (24)	1.1 (0.9–1.4)	81	47 (58)	1.1 (0.9–1.4)	201	97 (48)	1.4 (1.1–1.8)	502	44 (9)	3.8 (1.9–7.4)
Q4 (highest vulnerability)	786	194 (25)	1.2 (1.0–1.4)	31	13 (42)	0.8 (0.5–1.2)	135	59 (44)	1.2 (0.9–1.6)	620	122 (20)	8.4 (4.5–15.9)
**Social vulnerability related to household composition and disability**												
Q1 (lowest vulnerability)	786	240 (31)	Reference	228	115 (50)	Reference	215	103 (48)	Reference	343	22 (6)	Reference
Q2	786	163 (21)	0.7 (0.6–0.8)	122	70 (57)	1.1 (0.9–1.4)	181	66 (36)	0.8 (0.6–1.0)	483	27 (6)	0.9 (0.5–1.5)
Q3	784	181 (23)	0.8 (0.6–0.9)	58	33 (57)	1.1 (0.9–1.5)	190	98 (52)	1.1 (0.9–1.3)	536	50 (9)	1.5 (0.9–2.4)
Q4 (highest vulnerability)	786	163 (21)	0.7 (0.6–0.8)	28	9 (32)	0.6 (0.4–1.1)	144	58 (40)	0.8 (0.7–1.1)	614	96 (16)	2.4 (1.6–3.8)
**Social vulnerability related to racial and ethnic minority residents and English proficiency**												
Q1 (lowest vulnerability)	788	10 (1)	Reference	55	5 (9)	Reference	111	3 (3)	Reference	622	2 (0)	Reference
Q2	783	86 (11)	8.7 (4.5–16.5)	91	22 (24)	2.7 (1.1–6.6)	179	37 (21)	7.6 (2.4–24.2)	513	27 (5)	16.4 (3.9–68.5)
Q3	785	279 (36)	28.0 (15.0–52.2)	104	63 (61)	6.7 (2.8–15.6)	242	142 (59)	21.7 (7.1–66.6)	439	74 (17)	52.4 (12.9–212.4)
Q4 (highest vulnerability)	786	372 (47)	37.3 (20.1–69.3)	186	137 (74)	8.1 (3.5–18.8)	198	143 (72)	26.7 (8.7–81.9)	402	92 (23)	71.2 (17.6–287.3)
**Social vulnerability related to housing type and transportation**												
Q1 (lowest vulnerability)	786	87 (11)	Reference	159	70 (44)	Reference	139	14 (10)	Reference	488	3 (1)	Reference
Q2	786	149 (19)	1.7 (1.3–2.2)	112	57 (51)	1.2 (0.9–1.5)	158	60 (38)	3.8 (2.2–6.4)	516	32 (6)	10.1 (3.1–32.7)
Q3	785	218 (28)	2.5 (2.0–3.2)	87	52 (60)	1.4 (1.1–1.7)	219	117 (53)	5.3 (3.2–8.9)	479	49 (10)	16.6 (5.2–53.0)
Q4 (highest vulnerability)	785	293 (37)	3.4 (2.7–4.2)	78	48 (62)	1.4 (1.1–1.8)	214	134 (63)	6.2 (3.7–10.3)	493	111 (23)	36.6 (11.7–114.5)
**Individual components of social vulnerability related to racial and ethnic minority residents and English proficiency^§^**												
**Percentage of racial and ethnic minority residents (median = 16.1%)**												
Less than median	1,569	118 (8)	Reference	149	37 (25)	Reference	301	54 (18)	Reference	1,119	27 (2)	Reference
At or above median	1,567	629 (40)	5.3 (4.4–6.4)	287	190 (66)	2.7 (2.0–3.6)	429	271 (63)	3.5 (2.7–4.5)	857	168 (20)	8.1 (5.5–12.1)
**Percentage who speak English less than well (median = 0.7%)**												
Less than median	1,458	130 (9)	Reference	129	23 (18)	Reference	273	47 (17)	Reference	1,056	60 (6)	Reference
At or above median	1,684	617 (37)	4.1 (3.4–4.9)	307	204 (66)	3.7 (2.6–5.4)	457	278 (61)	3.5 (2.7–4.6)	920	135 (15)	2.6 (1.9–3.5)
**Individual components of social vulnerability related to housing type and transportation^§^**												
**Percentage of housing structures with ≥10 units (median = 2.9%)**												
Less than median	1,554	179 (12)	Reference	111	29 (26)	Reference	234	39 (17)	Reference	1,209	111 (9)	Reference
At or above median	1,588	568 (36)	3.1 (2.7–3.6)	325	198 (61)	2.3 (1.7–3.2)	496	286 (58)	3.5 (2.6–4.7)	767	84 (11)	1.2 (0.9–1.6)
**Percentage of housing units that are mobile home units (median = 10.9%)**												
Less than median	1,559	440 (28)	Reference	328	186 (57)	Reference	424	210 (50)	Reference	807	44 (5)	Reference
At or above median	1,583	307 (19)	0.7 (0.6–0.8)	108	41 (38)	0.7 (0.5–0.9)	306	115 (38)	0.8 (0.6–0.9)	1,169	151 (13)	2.4 (1.7–3.3)
**Percentage of households with more persons than rooms (median = 1.9%)**												
Less than median	1,513	235 (16)	Reference	213	88 (41)	Reference	350	112 (32)	Reference	950	35 (4)	Reference
At or above median	1,629	512 (31)	2.0 (1.8–2.3)	223	139 (62)	1.5 (1.2–1.8)	380	213 (56)	1.8 (1.5–2.1)	1,026	160 (16)	4.2 (3.0–6.0)
**Percentage of households without vehicle access (median = 5.7%)**												
Less than median	1,571	333 (21)	Reference	271	138 (51)	Reference	346	130 (38)	Reference	954	65 (7)	Reference
At or above median	1,571	414 (26)	1.2 (1.1–1.4)	165	89 (54)	1.1 (0.9–1.3)	384	195 (51)	1.4 (1.1–1.6)	1,022	130 (13)	1.9 (1.4–2.5)
**Percentage of persons living in institutionalized group quarters (median = 2%)**												
Less than median	1,569	348 (22)	Reference	273	149 (55)	Reference	334	122 (37)	Reference	962	77 (8)	Reference
At or above median	1,573	399 (25)	1.1 (1.0–1.3)	163	78 (48)	0.9 (0.7–1.1)	396	203 (51)	1.4 (1.2–1.7)	1,014	118 (12)	1.5 (1.1–1.9)

**Abbreviations:** CI = confidence interval; RR = risk ratio.

* Scores for all social vulnerability measures represented percentile rankings by county, ranging from 0–1, with higher scores indicating greater vulnerability. Scores were categorized into quartiles based on distribution among all U.S. counties.

^†^ Because of limited sample size, the National Center for Health Statistics urban/rural categories were collapsed into large metropolitan (which includes large central metropolitan and large fringe areas), medium and small metropolitan, and nonmetropolitan (micropolitan and noncore) areas.

^§^ Cutoffs for individual components of social vulnerability related to housing type and transportation were based on median values.

^¶^ P-values for Fisher’s exact tests yielded statistically significant findings (p<0.05) for all 95% CIs excluding the null value.

At the time of identification, incidence among new hotspot counties was 97 cases per 100,000 persons; in contrast, incidence in non-hotspot counties was 27 cases per 100,000 persons (p<0.001). Fourteen days later, hotspot county incidence was 140 cases per 100,000, and incidence in non-hotspot counties was 40 cases per 100,000 persons (p<0.001) (Figure 2). During the 14 days after hotspot detection, the absolute change in incidence in hotspot counties was higher than that in non-hotspot counties (p<0.001). Among hotspot counties, incidence was higher for counties with higher social vulnerability and particularly high in the highest quartile of social vulnerability on the day identified as a hotspot (212 cases versus 35–56 per 100,000 for other quartiles; p<0.001) and 14 days after being identified as a hotspot (234 cases versus 82–131 per 100,000; p<0.001) (Figure 2).

**FIGURE 2:**
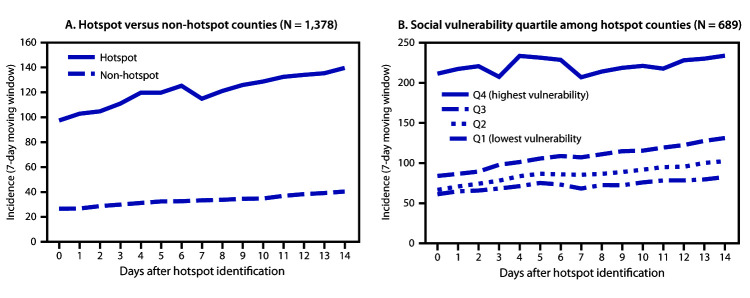
COVID-19 incidence* during the 14 days after identification as a hotspot, compared with counties not identified as hotspots^†^ (A) (N = 1,378 counties), and COVID-19 incidence, by quartile of social vulnerability index among hotspot counties^§^ (B) (N = 689 counties) — United States, June 1–July 25, 2020 * Cases per 100,000 persons; calculated based on 7-day moving window (total number of cases over the last 7 days per 100,000 population) during the 14 days after hotspot identification to smooth expected variation in daily case counts. ^†^ To compare incidence in hotspot and non-hotspot counties, a random sample of non-hotspot counties (1:1 ratio) was matched to hotspot counties by urbanicity and assigned the same date of reference. ^§^ Overall social vulnerability scores were percentile rankings ranging from 0–1, with higher values indicating more social vulnerability. Scores were categorized into quartiles based on distribution among all U.S. counties.

## Discussion

In this analysis, counties with more social vulnerabilities, particularly those with a higher percentage of racial and ethnic minority residents, high-density housing structures, and crowded housing units, were at higher risk for becoming a COVID-19 hotspot, especially in less urban areas. Among hotspot counties, areas with more social vulnerability had significantly higher incidence than did other counties. These findings have implications for efforts to prevent counties with social vulnerability from becoming COVID-19 hotspots, including prioritizing vaccination access,*** and for implementing public health action in counties that become hotspots.

Consistent with previous findings (*4*–*6*), these results show that COVID-19 disproportionately affects racial and ethnic minority groups, who might also experience more socioeconomic challenges.^†††^ Communities with higher social vulnerability have a higher percentage of racial and ethnic minority residents, who might be more likely to have essential jobs requiring in-person work and live in potentially crowded conditions (*7*,*8*). These circumstances could put racial and ethnic minority residents at risk for COVID-19 through close contact with others. Incorporating the needs of populations that are socially vulnerable into community mitigation plans is essential for limiting COVID-19 transmission. Specifically, implementing recommended prevention efforts at facilities requiring in-person work (e.g., meat processing facilities and grocery stores), including temperature or symptom screening, mask mandates, social distancing practices, and paid sick leave policies encouraging ill workers to remain home, might reduce transmission risk among populations that are vulnerable at workplaces (*9*). In addition, plain-language and culturally sensitive and relevant public health messaging should be tailored based on community needs, communicated by local leaders, and translated into other languages in areas with many nonnative English speakers (*9*).

Additional support from federal, state, and local partners is needed for communities with social vulnerabilities and at risk for COVID-19, particularly for persons living in crowded or high-density housing conditions. Initiatives to provide temporary housing, food, and medication for COVID-19 patients living in crowded housing units could be considered to permit separation from household members during infectious periods.^§§§^

As expected, hotspot counties had significantly higher COVID-19 incidence at the time of detection than did non-hotspot counties. Hotspot counties also had a higher absolute change in incidence during the 14 days after identification, demonstrating real and meaningful increases in incidence in these counties and underscoring the importance of implementing robust public health responses in these counties. Among hotspot counties, areas with the highest social vulnerability had significantly higher incidence, indicating an urgent need to prioritize public health action in these counties to curb COVID-19 transmission. Hotspot data informed deployment of multiagency response teams from CDC, the Federal Emergency Management Agency, the Office of the Assistant Secretary for Preparedness and Response, and the Office of the Associate Secretary for Health, to 33 locations in 21 states during June 29–July 24. These COVID-19 Response Assistance Field Teams (CRAFTs) learned from state and local leaders about local response efforts and assessed how federal assistance could augment local efforts to reduce the impact of the COVID-19 pandemic. Areas with high social vulnerability need continued support in developing and implementing mitigation strategies and strengthening contact tracing programs to quickly identify and isolate COVID-19 cases and limit transmission.

The findings in this report are subject to at least three limitations. First, associations between social vulnerability and risk for COVID-19 infection using person-level data could not be assessed; it was also not possible to assess confounding by factors such as employment. Second, changes in testing availability and laboratory reporting might have affected COVID-19 incidence estimates and hotspot detection. Finally, the hotspot criteria might have limited the ability to detect hotspots in counties with smaller populations.

Building on previous work (*10*), these findings underscore the need for federal, state, and local partners to work with community leaders to support areas with high social vulnerability and prevent them from becoming COVID-19 hotspots. These findings also demonstrate the need to reevaluate factors related to high incidence for earlier detection of hotspot counties, particularly in areas with high social vulnerabilities; among hotspot counties, these results demonstrate the need to prioritize immediate public health action in counties with the highest social vulnerability, especially in less urban areas.

**Summary**

**What is already known about this topic?**

Communities with higher social vulnerabilities, including poverty and crowded housing units, have more adverse outcomes during and following a public health event.

**What is added by this report?**

Counties with greater social vulnerability were more likely to become areas with rapidly increasing COVID-19 incidence (hotspot counties), especially counties with higher percentages of racial and ethnic minority residents and people living in crowded housing conditions, and in less urban areas. Hotspot counties with higher social vulnerability had high and increasing incidence after identification.

**What are the implications for public health practice?**

Focused public health action is urgently needed to prevent communities that are socially vulnerable from becoming COVID-19 hotspots and address persistently high COVID-19 incidence among hotspot areas that are socially vulnerable.

## Footnotes

* https://usafacts.org/issues/coronavirus.

† Areas defined as hotspot counties met all four of the following criteria, relative to the date assessed: 1) >100 new COVID-19 cases in the most recent 7 days, 2) higher COVID-19 incidence in the most recent 7 days incidence compared with the preceding 7 days, 3) a decrease of <60% or an increase in the most recent 3-day COVID-19 incidence over the preceding 3-day incidence, and 4) the ratio of 7-day incidence to 30-day incidence exceeds 0.31. In addition, hotspots must have met at least one of the following criteria: 1) >60% change in the most recent 3-day COVID-19 incidence or 2) >60% change in the most recent 7-day incidence. CDC and other federal agencies that are monitoring trends in COVID-19 are collaborating to refine approaches to define and monitor hotspots. As a result, terminology or definitions used in future reports might differ from the terminology used in this report.

§ The 15 population-based social factors incorporated into the SVI measures were four domains: 1) socioeconomic status, which was based on poverty, employment, income, and educational attainment; 2) household composition and disability, which was based on age (pediatric and elderly populations), civilians aged >5 years with a disability, and single-parent households; 3) racial and ethnic minority residents (i.e., do not identify as White, non-Hispanic/Latino) and English proficiency, which was based on representation of racial and ethnic minority residents and English proficiency; and 4) housing type and transportation, which was based on multiunit structures, mobile homes, crowding, no household vehicle access, and institutionalized group quarters. https://www.atsdr.cdc.gov/placeandhealth/svi/index.html.

¶ According to the 2013 National Center for Health Statistics Urban-Rural Classification Scheme for counties, counties can be categorized into one of six categories based on population size, including large central metropolitan, large fringe metropolitan, medium metropolitan, small metropolitan, micropolitan, and noncore areas. For this analysis, results were presented in three categories: large central metropolitan and large fringe metropolitan (large metropolitan); medium and small metropolitan; and micropolitan and noncore areas (nonmetropolitan).

** P-values for Fisher’s exact tests were used to determine statistical significance.

†† New hotspot counties met hotspot criteria after ≥21 days of not meeting hotspot criteria. This component of the analysis was limited to new hotspot counties to understand trends after initial hotspot identification.

§§ Incidence was calculated based on 7-day moving window during the 14 days after hotspot identification to smooth expected variation in daily case counts.

¶¶ For incidence comparisons, statistically significant differences were evaluated using the Wilcoxon rank-sum test.

***
https://www.nap.edu/catalog/25917/framework-for-equitable-allocation-of-covid-19-vaccine#resources .

†††
https://www.cdc.gov/coronavirus/2019-ncov/community/health-equity/race-ethnicity.html#fn19 .

§§§
https://covid19.ca.gov/housing-for-agricultural-workers/ .
